# Symptoms provide predictive information on daily functioning beyond signs and diseases in middle-aged and older adults, particularly those with multimorbidity

**DOI:** 10.1093/ageing/afag046

**Published:** 2026-03-02

**Authors:** Geeske Peeters, Cillian Hourican, Mike Lees, Rick Quax, René Melis, Almar Kok, Natasja M van Schoor, Marcel Olde Rikkert

**Affiliations:** Department of Geriatric Medicine, Radboud University Medical Center, Nijmegen 6500 HB, The Netherlands; Radboudumc Alzheimer Center, Radboud University Medical Center, Nijmegen 6500 HB, The Netherlands; Institute of Informatics, University of Amsterdam, Amsterdam, The Netherlands; Institute of Informatics, University of Amsterdam, Amsterdam, The Netherlands; Institute of Informatics, University of Amsterdam, Amsterdam, The Netherlands; Institute for Advanced Study, University of Amsterdam, Amsterdam, The Netherlands; Department of Geriatric Medicine, Radboud University Medical Center, Nijmegen 6500 HB, The Netherlands; Radboudumc Alzheimer Center, Radboud University Medical Center, Nijmegen 6500 HB, The Netherlands; Department of Epidemiology and Data Science, Amsterdam UMC location Vrije Universiteit Amsterdam, Amsterdam, The Netherlands; Department of Psychiatry, Amsterdam UMC Location Vrije Universiteit Amsterdam, Amsterdam, The Netherlands; Amsterdam Public Health Research Institute, Amsterdam UMC Locatie VUmc—Epidemiology and Data Science, Amsterdam, Noord-Holland, The Netherlands; Department of Geriatric Medicine, Radboud University Medical Center, Nijmegen 6500 HB, The Netherlands; Radboudumc Alzheimer Center, Radboud University Medical Center, Nijmegen 6500 HB, The Netherlands

**Keywords:** frailty, comorbidity, functional limitations, chronic disease, symptom burden, older people

## Abstract

**Background:**

In people with multimorbidity, traditional, disease-oriented approaches may overlook the impact of symptoms on daily functioning.

**Objective:**

To explore the assumption that symptoms and signs provide information on functional limitations beyond that of diseases in older adults, specifically those with multimorbidity.

**Subjects:**

4025 participants in the Longitudinal Aging Study Amsterdam (1995–2022).

**Methods:**

Analyses included six symptoms, five signs and eight diseases as exposures and a sum score of six functional limitations as the outcome. Partial Information Decomposition was used to partition the total variability in functional limitations into unique, redundant and synergistic information provided by the exposures in the total sample and in the multimorbidity subgroup. Random forest prediction models were run to examine the added predictive value of symptoms, signs and diseases.

**Results:**

In the total sample, 59% had multimorbidity. Symptoms, signs and diseases together explained 13.3% of variability in functional limitations. None of the three domains contributed unique information. Synergy accounted for most of the explained variability (signs = 9.2%, symptoms = 34.3%, diseases = 34.3%). In the multimorbidity subgroup, symptoms, signs and diseases together explained 8.7% of variability in functional limitations. Symptoms uniquely contributed 35.5% of their information, while signs and diseases were redundant. Prediction models showed that symptoms provided substantial predictive value beyond diseases alone, with a 110% increase in predictive agreement when symptoms were added to diseases in the multimorbidity subgroup, compared to 58% in the total sample.

**Conclusions:**

In people with multimorbidity, symptoms and signs explain more variability in functional limitations than diseases alone, supporting the need for a symptom-oriented approach in clinical care and research.

## Key Points

- In people with multimorbidity, but not in the general population, the six measured symptoms provide unique information on a sum score of six functional limitations that is not provided by the eight diseases or five signs.
- The finding that symptoms explain more of the variation in functional limitations than diseases and signs was confirmed by two distinct statistical approaches.
- The findings support the need for a symptom-oriented approach in clinical care and research in the context of multimorbidity.

## Introduction

The prevalence of multimorbidity, i.e. having two or more coexisting chronic diseases, increases with age from approximately 10%–30% at the age of 40 to 50%–85% at the age of 75 [1, 2]. Multimorbidity is associated with declines in functioning and quality of life [3, 4] and high costs of care [5]. In the absence of a cure, care focuses on symptom management. Clinical guidelines traditionally use a single disease approach [6], and clinicians typically evaluate effects of treatment by monitoring signs (e.g. blood pressure for hypertension and glucose levels for diabetes). However, in the context of multimorbidity, parallel treatment of single diseases can lead to high treatment burden, overtreatment, adverse interactions between therapies and subsequently suboptimal, fragmented care [7, 8]. Multimorbidity requires a more holistic, integrated care approach. Beyond diseases (as diagnostic labels) and signs (objectively measured manifestations of diseases), symptoms (subjectively experienced manifestations of diseases) shape the patient’s experience and disease trajectory. Focusing on symptom relief may help to adopt a more holistic approach that better aligns with the patient’s experience.

In the context of multimorbidity, a single symptom may be caused by multiple diseases or interactions between these diseases. For example, thyroid disorder is a common comorbidity in heart failure and both diseases, independently and jointly, can cause fatigue [9]. Moreover, symptoms may trigger new symptoms independent of underlying diseases, resulting in atypical disease presentations. For example, in frail older adults, COVID-19 often presents with a fall as a consequence of reduced attention [10]. Atypical disease presentations are more common in (frail) older adults [11] and challenge the common conception that (changes in) symptoms are mere presentations of new or exacerbating diseases. Atypical symptom behaviour aligns with the idea that health is an adaptive state that emerges from interactions between social and biological factors [12]. This suggests that symptoms may behave partly independently of disease activity. Thus, symptoms may carry information that is insufficiently captured by diagnostic disease labels. The observation that symptoms trigger other symptoms illustrate the necessity to look at symptoms collectively (rather than individually) and to account for potential interactions between symptoms [13].

If indeed a symptom-oriented approach is appropriate (complementary to the disease-oriented approach) in the clinical care for, and research of, older adults with multimorbidity, then a new paradigm is required. The assumption underlying such a symptom-oriented approach is that symptoms and signs provide information beyond that of diseases in people with multimorbidity. The aim in this paper is to test that assumption. We hypothesise that symptoms and signs provide predictive information on functional limitations beyond that of diseases in people with multimorbidity. While we expect this hypothesis to be true specifically in the context of multimorbidity, we examine this assumption also in the general community-dwelling middle-aged and older population to verify this.

## Methods

### Study design and participants

Data were used from participants in the Longitudinal Aging Study Amsterdam (LASA), an ongoing population-based cohort study [14, 15]. The original cohort of 3107 community-dwelling older adults aged 55–85 years was recruited in 1992/93 with triennial follow-up. The sample was replenished with participants aged 55–65 years in 2002/03 (n = 1002) and 2012/13 (n = 1023). Data were collected via face-to-face structured interviews and self-completed questionnaires covering physical, cognitive, emotional and social domains. The study was approved by the Medical Ethics Committee of the VU University Medical Center Amsterdam (file numbers: 92/138, 2002/141, 2012/361, 2016.301). All participants signed informed consent.

For the current analyses, we used data from the 1995/96 through 2021/22 waves. Participants with more than 50% missing values across all relevant variables in a certain wave, were excluded from the analyses for that wave. Missing values were imputed using iterative random forest procedures (Multiple Imputation by Chained Equations, 40 iterations, max depth = 5, minimum samples per leaf = 10, n estimators = 100) [16, 17]. After imputation, the analytic dataset comprised 14,717 observations from 4025 participants across all waves.

### Symptoms, signs and diseases

In this paper, ‘diseases’ refers to the diagnostic labels of abnormal conditions that affect the structure or function of part or all of the body and usually are associated with specific signs and symptoms [18]; ‘signs’ refers to objectively measured manifestations of diseases; and ‘symptoms’ refers to subjectively experienced manifestations of diseases. Selection of diseases, symptoms and signs was based on (i) availability of data for the whole sample (ruling out symptoms measured in subgroups with a certain disease diagnosis); (ii) and consistent measurement across waves to be able to include as many waves as possible. Six symptoms and five signs were measured and dichotomised using standardised questionnaires and cut-points as described in the Supplemental Data File, Appendix 1. Symptoms included depressive symptoms, anxiety symptoms, sleep problems, pain and hearing complaints. Signs included mean arterial pressure, grip strength, BMI, waist-hip-ratio and cognitive functioning. Eight chronic diseases were measured as self-reported diagnosed diseases, including chronic nonspecific lung disease, cardiac disease, peripheral arterial disease, diabetes mellitus, stroke, osteoarthritis, rheumatoid arthritis and cancer. The number of present symptoms (0–6), signs (0–5) and diseases (0–8) were summed.

### Functional limitations

Functional limitations were assessed using six self-reported items on limitations in daily activities, including stair use, dressing, cutting toenails, walking outside, sitting/rising from a chair and transportation. These items were derived from the 6-item Capacity for Self-Care indicator and the 9-item Organisation for European Cooperation and Development (OECD) indicator, with item selection based on the items with the highest item-rest correlation [19]. Each item was rated on a five-point ordinal scale from 0 (‘Yes, without difficulty’) to 4 (‘No, I cannot’). Responses were summed to create a total functional limitations score (range 0–24, higher scores indicate more limitations).

### Statistical analyses

To quantify how symptoms, signs and diseases individually and jointly relate to functional limitations, we require a multivariate correlation measure that can capture nonlinear relationships among categorical data, and a method to decompose these correlations. Therefore, we used mutual information, where the decomposition was done by Partial Information Decomposition (PID) using the Minimum Mutual Information (MMI) metric [20]. Mutual information quantifies the statistical dependence of the outcome variable (functional limitations) on the explanatory variable (e.g. symptoms). Mutual information is a nonparametric method that deals with nonlinear and complex relationships between categorical variables. PID decomposes total mutual information, expressed as ‘bits’ of information, into its constituent parts, i.e. unique, redundant, lost and synergistic information. ‘Unique information’ reflects the predictive contribution of a single explanatory variable independently of the others. For example, if a symptom provides zero unique information, this indicates that its predictive value is either entirely shared with other variables or only emerges when combined with them. ‘Redundant information’ represents the shared predictive insights about the outcome variable that multiple explanatory variables offer (e.g. the overlapping information conveyed by both signs and symptoms). ‘Lost information’ captures the information lost if we exclude a specific exposure. ‘Synergistic information’ captures the added value that arises when explanatory variables are considered together. It quantifies how a combination of, say, signs and symptoms can predict functional limitations more accurately than the sum of their individual contributions.

To ensure a balanced distribution in each of the variables while preserving interpretability and ordinal structure, the continuous functional limitations score (0–24) was categorised as: no, 1, 2–5 and 6–24 limitations (based on approximate tertiles for scores > 0). Sign and disease counts were categorised into four discrete groups: 0, 1, 2, and ≥3. The symptom count was categorised as 0–1, 2, 3, and ≥4.

To verify that our results are not specific to one methodological approach, and to specifically examine the added predictive value of symptoms to signs and diseases, we additionally ran random forest (RF) models with 100 trees to predict functional limitations. Rather than using sum scores, we included each symptom, sign and disease as a separate explanatory variable to make optimal use of the available information. Functional limitations were included as a discrete variable (range 0–30). A series of models were run, adding all symptoms, all signs and all diseases step by step: first starting with diseases, adding signs and symptoms; then starting with symptoms, adding signs and diseases. Model performance was quantified as the agreement between observed and predicted functional limitations using quadratic-weighted Cohen’s κ.

Both the PID and RF models were run on the total sample and separately in the multimorbidity subgroup. We are primarily interested in the group with multimorbidity. The results in the total sample provide a reference for the interpretation of the results in the group with multimorbidity.

To optimise computational power needed for the PID and RF models, data from waves 1995/96 to 2021/21 were merged and treated as one ‘cross-sectional’ dataset. The models do not account for the dependence of observations within persons over time, which may result in inflated or skewed results. Sensitivity analyses were done to examine the potential influence of ignoring this dependence in observations by repeating the RF models using data from wave 1995/96 only and wave 2021/22 only (first and last waves).

## Results

Across all waves, the 4025 participants were on average 73 ± 8 years of age (range 57–102), 46% were women and 59% had multimorbidity. Participants had a median (interquartile range (IQR)) count of 2 (2–3) symptoms, 1 (1–2) signs and 1 (1–2) diseases. The median (continuous) number of functional limitations was 1 (0–4).

In the total sample, total mutual information from symptoms, signs and diseases was 0.252 bits, explaining 13.3% of the variance in functional limitations (Table 1). This was almost entirely interaction-driven: none of the three domains contributed unique information, and pure synergy accounted for most of the explained variance (signs = 9.3%, symptoms = 34.7%, diseases = 34.7%). Direct mutual information with the target was modest (symptoms = 7.1%, diseases = 6.7%, signs = 1.6%), underscoring the dominance of cross-domain interactions. Removing any single domain led to similar information loss for symptoms (35.4%) and diseases (34.7%), with a smaller loss for signs (9.3%). Sensitivity analyses repeating the PID models using waves 1995/96 and 2021/22 data only, showed roughly similar patterns in the results (Supplemental Data File, Appendix 2), with information explained by symptoms, signs and diseases being predominantly interaction-driven.

**Table 1:** Variability in functional limitations explained by symptoms, signs and diseases.

Domain	Lost information	Unique information	MI with functional limitations	Pure synergy	Synergy bounds (%)
Total sample: entropy = 1.889, total MI = 0.252					
Signs	9.31% (0.023 bits)	0	1.60% (0.030 bits)	9.31% (0.023 bits)	9.31%–17.57% (0.023–0.044 bits)
Symptoms	35.39% (0.089 bits)	0	7.10% (0.134 bits)	34.67% (0.087 bits)	35.39%–42.93% (0.089–0.108 bits)
Diseases	34.67% (0.087 bits)	0	6.7% (0.144 bits)	34.67% (0.087 bits)	34.67%–42.93% (0.087–0.108 bits)
Multimorbidity subgroup: entropy = 1.991, total MI = 0.169					
Signs	14.90% (0.025 bits)	0	1.13% (0.022 bits)	14.90% (0.025 bits)	14.90%–29.77% (0.025–0.050 bits)
Symptoms	64.76% (0.109 bits)	34.13% (0.058 bits)	5.87% (0.117 bits)	15.77% (0.027 bits)	30.63%–30.63% (0.052–0.052 bits)
Diseases	15.77% (0.027 bits)	0	1.98% (0.039 bits)	15.77% (0.027 bits)	15.77%–30.63% (0.027–0.052 bits)

Mutual information (MI) = quantifies how much variability in the outcome functional limitations is captured by information on symptoms, signs and diseases. Total mutual information captures the combined predictive power of all three domains. In the total sample, this is 0.252 bits of information, equalling 13.3% of the total variability in functional limitations (entropy = 1.889 bits = 100%). In the multimorbidity subgroup, this is 0.169 bits, equalling 8.5% of the variability (entropy = 1.991 bits = 100%). Lost information = captures the information lost if we exclude a specific exposure. Unique information = refers to the amount of predictive power that a single domain (signs, symptoms or diseases) contributes on its own, independent of the other domains. If unique information is zero, it means every insight a given domain offers about functional limitations can be gleaned equally from other domains or only arises in combination with them. Synergy lower bound = the guaranteed, minimal extra information from combinations involving that domain—essentially, what you definitely lose if you never allow it to interact with any other domain. Computed as the lost information minus the unique information. Synergy upper bound = the maximal possible extra signal that could come from any combination of that domain with one, two or all three of the other domains. It shows the ceiling on how much interaction-driven information that domain might contribute. Percentages indicate the relative contribution of each domain in explaining functional limitations compared to the total explained variance (entropy = 1.889 bits = 100%). Higher percentages indicate a larger contribution.

Compared with the total sample (0.252 bits, 13.3% of variance), in participants with multimorbidity, total information was lower: 0.169 bits (8.5% of variance) (Table 1). Symptoms now provided 34.1% unique information and omitting them forfeited 64.8% of explanatory power. Diseases and signs provided no unique information and far smaller losses on removal (15.8% and 14.9%, respectively). Pure symptom and disease synergy halved to 15.8%, whereas synergy from signs increased to 14.9%, indicating that signs contributed chiefly through interactions. Direct mutual information likewise declined (symptoms = 5.9%, diseases = 2.0%, signs = 1.1%), showing that, in multimorbidity participants, functional limitations are driven more by symptom burden, with disease and signs adding little information beyond symptoms. Sensitivity analyses showed mostly similar results except that in wave 2021/22., symptoms did not provide unique information (Supplemental Data File, Appendix 2). In both waves, pure synergy was higher for all domains, suggesting that a significant proportion of the information is contributed through interactions.

Similar to the results in the PID models, the RF models showed that adding symptoms to diseases produced substantial improvements in predictive performance (Table 2). In the total sample, the agreement improved minimally after adding signs (κ = 0.354) and substantially after adding symptoms (κ = 0.509), resulting in a 58% improvement from the disease-only model (κ = 0.310). In the multimorbidity group, the agreement improved 110% after adding signs (κ = 0.287) and symptoms (κ = 0.460) to the disease only model (κ = 0.208). Vice versa, the gain in agreement was much smaller when diseases and signs were added to symptom only models (Table 2). Sensitivity analyses repeating the RF models using waves 1995/96 and 2021/22 data only, showed similar results, but with wider confidence intervals: the agreement improved more when symptoms were added to diseases or signs than when diseases or signs were added to symptoms (Supplemental Data File, Appendix 2).

**Table 2:** Results of the prediction models estimating the additional predictive value of symptoms, diseases and signs beyond that of the other domains.

	Diseases only, mean Cohen’s κ (CI)	Diseases + signs, mean Cohen’s κ (CI)	Diseases + signs + symptoms, mean Cohen’s κ (CI)
Total sample	0.310 (0.283–0.337)	0.354 (0.325–0.381)	0·511 (0·487–0·536)
Multimorbidity subgroup	0.208 (0.172–0.230)	0.287 (0.259–0.315)	0·459 (0·422–0·489)
	Symptoms only, mean Cohen’s κ (CI)	Symptoms + signs, mean Cohen’s κ (CI)	Symptoms + signs + diseases, mean Cohen’s κ (CI)
Total sample	0.440 (0.416–0.458)	0.465 (0.438–0.488)	0·511 (0·487–0·536)
Multimorbidity subgroup	0.386 (0.358–0.416)	0.413 (0.385–0.445)	0·459 (0·422–0·489)
	Signs only, mean Cohen’s κ (CI)	Signs + symptoms, mean Cohen’s κ (CI)	Signs + symptoms + diseases, mean Cohen’s κ (CI)
Total sample	0.084 (0.068–0.101)	0·466 (0·441–0·491)	0·511 (0·487–0·536)
Multimorbidity subgroup	0.106 (0.086–0.128)	0·414 (0·387–0·445)	0·459 (0·422–0·489)

The mean Cohen’s κ and its 95% confidence intervals (CI) expresses the agreement between observed and predicted functional limitations, with higher agreement indicating more accurate prediction.

## Discussion

Taken together, the information-theoretic and predictive analyses present a coherent picture. In the total sample, signs, symptoms and diseases show considerable overlap in the information they provide on functional limitations (i.e. high redundancy), while also contributing synergistic information. Notably, symptoms offer a substantial predictive advantage beyond what diseases alone can explain. Similar patterns emerged in the multimorbidity subgroup, with the key distinction that symptoms contributed more unique information in this group. These findings support the view that, in individuals with multimorbidity, symptoms convey important information beyond what is captured by disease diagnoses alone.

Findings from both models indicate that symptoms capture unique predictive information for daily functioning not contained in disease diagnoses, while signs provide minimal additional benefit when added to diseases. This suggests that symptoms reflect distinct aspects of daily functioning not captured by signs or diseases. While the mutual information was higher in the total sample (13.3%) than in the multimorbidity subgroup (8.5%), the relative contribution of symptoms was more pronounced in the multimorbidity subgroup, supporting the idea that symptoms may behave partly independently of disease activity in people with multimorbidity. In people with a single disease, the link between symptoms and diseases is more direct, whereas in the context of multimorbidity, a single symptom can be caused by multiple diseases and symptoms can trigger new symptoms. For example, both joint stiffness and poor eyesight can lead to a fall, which can lead to pain. This pain is not directly linked to the eye disease that causes the poor eyesight or osteoarthritis causing the stiffness. Clinical reasoning based on differential diagnoses often focuses on single disease causality, which is not the best reasoning in the context of multimorbidity, as shown here. The findings support the idea that in the context of multimorbidity, physicians should look at the full constellation of symptoms, signs and diseases to understand the drivers of limitations in daily functioning, rather than attributing functional impairments to individual diseases, symptoms or signs.

The finding that symptoms, signs and diseases provide synergistic information implies that their combination tells us more about daily functioning than the sum of the information provided by each variable. This supports the logic of deficits-based definitions of frailty [21], which posit that frailty arises not from any single deficit, but from the accumulation and ‘interaction’ of multiple deficits—typically a mix of diseases and functional limitations [21]. That is, frailty is not captured by individual deficits but appears as an emergent state in the presence of multiple deficits and their interactions.

While the current findings may be intuitive to clinicians and physicians working in geriatrics and rehabilitation, we were surprised to find little direct evidence for this in the literature. Our findings align with the few other studies that decomposed the shared and unique contributions of symptoms, signs and diseases. Previous analyses in the same cohort showed that disease-specific symptoms provide more detailed information about the impact of chronic diseases on mobility, than diseases alone [22]. A study among 1073 HIV patients showed that adding signs and symptoms to multimorbidity patterns changed the strength of associations with patient reported outcomes [23]. To examine robustness of our findings, we recommend validation in other cohorts and varying settings, particularly clinical settings.

Key strengths of this study include the large, well-characterised LASA cohort including middle-aged and older adults with long-term follow-up, and the dual use of information theory and predictive RF models. In a large study such as LASA, it is impossible to consistently measure all potentially relevant symptoms, signs and diseases. However, the symptoms, signs and diseases that were measured were selected based on their high prevalence and relevance for an ageing community-dwelling population. Moreover, severity of symptoms and signs was not taken into account. This information was available for some but not all items. We therefore decided to treat all items equally by dichotomising symptoms and signs as present or not present and using sum scores. However, it is likely that incorporating severity would result in stronger associations with functional limitations. Further limitations include reliance on self-reported symptoms and diseases and the necessary discretization for PID, which may mask subtle effects. Continuous-variable decompositions may provide more precise estimates of the unique and shared contributions of each explained variable, although this is unlikely to lead to a different conclusion.

In conclusion, our findings confirm the hypothesis that symptoms provide information beyond that of diseases, particularly in people with multimorbidity. If these findings are verified in in other cohorts and settings, these findings support the idea that a symptom-oriented approach may add value beyond that of a disease-oriented approach in multimorbidity care and research. Physicians are encouraged to look at the full constellation of symptoms, signs and disease to understand drivers of limitations in daily functioning.

## Supplementary Material

aa_-_25_-_2487_-_File002_afag046

## Data Availability

Data from LASA are available for use for specific research questions, provided that an agreement is made up. Research proposals should be submitted to the LASA Steering Group, using a standard analysis proposal form that can be obtained from the LASA website: www.lasa-vu.nl. Files with data published in this publication are freely available for replication purposes and can be obtained using the same analysis proposal form. The LASA Steering Group will review all requests for data to ensure that proposals for the use of LASA data do not violate privacy regulations and are in keeping with informed consent that is provided by all LASA participants.
