# **Correction to:** Symptoms provide predictive information on daily functioning beyond signs and diseases in middle-aged and older adults, particularly those with multimorbidity

**DOI:** 10.1093/ageing/afag278

**Published:** 2026-09-12

This is a correction to: Geeske Peeters, Cillian Hourican, Mike Lees, Rick Quax, René Melis, Almar Kok, Natasja M van Schoor, Marcel Olde Rikkert, Symptoms provide predictive information on daily functioning beyond signs and diseases in middle-aged and older adults, particularly those with multimorbidity, *Age and Ageing*, Volume 55, Issue 2, February 2026, afag046, https://doi.org/10.1093/ageing/afag046

In the originally published version of this manuscript, the statement of equal contribution and specific contributions for Cillian Hourican and Geeske Peeters was inadvertently omitted.

The **Acknowledgements** section should read as follows:

**Acknowledgements:**

Hourican and Peeters contributed equally to this paper. Hourican and Peeters were responsible for the study design. Hourican was responsible for analyses and presentation of the results. Peeters drafted the manuscript. All other authors contributed to interpretation of the findings, provided feedback and approved the final draft.

