# Supplementary material for: Symptoms provide predictive information on daily functioning beyond signs and diseases in middle-aged and older adults, particularly those with multimorbidity
Source: Age Ageing. 2026 Mar 2;55(2):afag046. doi: 10.1093/ageing/afag046 (PMC13570245; doi:10.1093/ageing/afag046)
Supplement: Supplementary file 1 [file aa_-_25_-_2487_-_file002_afag046.docx]

**Symptoms provide predictive information on daily functioning beyond signs and diseases in middle-aged and older adults, particularly those with multimorbidity**

**Supplemental Data File**

**Content**

Appendix 1 - Expanded methods: description of the methods used to measure the	p. 2

symptoms and signs in LASA

Appendix 2 - Sensitivity analyses repeating the PID models using data from waves	p. 4

1995/96 and 2021/22

Appendix 3 - Sensitivity analyses repeating the Random Forest models using data	p. 6

from waves 1995/96 and 2021/22

**Appendix 1**

**Expanded methods: description of the methods used to measure the symptoms and signs in LASA**

The six symptoms included:

- Depressive symptoms was assessed with the Center for Epidemiologic Studies Depression Scale (CES‑D), with scores of 16 and higher indicating presence of depressive symptoms [1].
- Anxiety symptoms were identified using the anxiety subscale of the Hospital Anxiety and Depression Scale (HADS), with scores of 8 or greater indicating presence of clinically relevant anxiety symptoms [2].
- Sleep problems (range 3-12) was derived from three categorical questions asking about problems falling asleep, waking through the night and waking up too early. Sleep problems were present if participants scored positively on at least one of these questions.
- Pain severity was measured using a subscale of the Nottingham Health Profile (range 5-10) and dichotomized as score of 5 (no pain) versus >5 (pain present) [3].
- Hearing complaints were coded as present if any difficulty or inability to hear was reported.
- Memory complaints were determined by a yes/no response to the question: ‘‘Do you have complaints about your memory?’’.

The five signs included:

- Mean arterial pressure (MAP) was calculated as the average of the diastolic and systolic blood pressure measured with the automatic Omron device (Omron HEM 815F). MAP was dichotomized using a threshold of 136.5 mmHg, representing the midpoint between normal values and the European guideline–defined hypertension cutoff of 170 mmHg [4].
- Grip strength was measured using a hand grip dynamometer (Takei TKK 5001, Takei Scientific Instruments Co. Ltd). Sex‑specific thresholds (<27 for men, <16 for women) established by the EWGSOP2 consensus were used to identify individuals with low muscle strength [5].
- Body mass index (BMI) was calculated based on measured height and weight and dichotomized as obese (BMI>30) vs. non‑obese (BMI<30) [6].
- The waist‑hip ratio (WHR) was calculated as the waist circumference divided by hip circumference measured using standardized procedures. WHR was dichotomised using sex-specific cutoffs of ≥ 0.90 in men and ≥ 0.85 in women to indicate abdominal obesity [7].
- Cognitive functioning was measured with the Mini‑Mental State Examination (MMSE), with scores of 23 or lower indicating impairment [8].

Appendix 2. Sensitivity analyses repeating the PID models using data from waves 1995/96 and 2021/22

| Domain | Lost Information | Unique Information | MI with Functional Limitations | Pure Synergy | Synergy Bounds (%) |
| --- | --- | --- | --- | --- | --- |
| **Total sample** | | | | | |
| Wave 1995/96: entropy=1.842, total MI=0.301 | | | | | |
| Signs | 19.34%  (0.058 bits) | 0% | 1.50%  (0.028 bits) | 19.34%  (0.058 bits) | 9.34 - 29.74%  (0.058 - 0.090 bits) |
| Symptoms | 48.14%  (0.0.145 bits) | 0.37 %  (0.001 bits) | 8.54%  (0.157 bits) | 37.37% (0.112 bits) | 47.78 - 47.78%  (0.144 - 0.144 bits) |
| Diseases | 37.37%  (0.112 bits) | 0% | 7.02%  (0.129 bits) | 37.37%  (0.112 bits) | 37.37 - 47.78%  (0.112 - 0.144 bits) |
| Wave 2021/22: entropy=1.901, total MI=0.349 | | | | | |
| Signs | 28.80%  (0.101 bits) | 0% | 2.79 %  (0.053 bits) | 28.80%  (0.101 bits) | 28.80 – 45.71%  (0.101 - 0.159 bits) |
| Symptoms | 40.12%  (0.140 bits) | 0% | 6.41%  (0.122 bits) | 40.12% (0.140 bits) | 40.12 – 57.03%  (0.140 - 0.199 bits) |
| Diseases | 46.48%  (0.162 bits) | 0% | 7.89%  (0.150 bits) | 40.12%  (0.140 bits) | 46.48 – 57.03%  (0.162 - 0.199 bits) |
| **Multimorbidity subgroup** | | |  |  |  |
| Wave 1995/96: entropy=1.972 , MI=0.277 | | | | | |
| Signs | 28.73%  (0.080 bits) | 0% | 1.16%  (0.023 bits) | 28.73% (0.080 bits) | 28.73% - 40.21%  (0.080 - 0.111 bits) |
| Symptoms | 69.41%  (0.192 bits) | 28.88%  (0.080 bits) | 8.35% (0.165 bits) | 28.73%  (0.080 bits) | 40.21% - 40.21%  (0.111 - 0.111 bits) |
| Diseases | 23.29%  (0.064 bits) | 0% | 2.67%  (0.053 bits) | 23.29%  (0.064 bits) | 23.29% - 35.12%  (0.064 - 0.097 bits) |
| Wave 2021/22: entropy=1.997 , MI=0.294 | | | | | |
| Signs | 39.83%  (0.117 bits) | 0% | 4.29%  (0.086 bits) | 39.83% (0.117 bits) | 39.83% - 58.91%  (0.117 - 0.0.173 bits) |
| Symptoms | 52.76%  (0.155 bits) | 0% | 6.06%  (0.121 bits) | 39.83%  (0.117 bits) | 52.76% - 58.91%  (0.156 - 0.173 bits) |
| Diseases | 23.75%  (0.070 bits) | 0% | 3.34%  (0.007 bits) | 23.75%  (0.070 bits) | 23.75% - 42.84%  (0.070 - 0.126 bits) |

*Mutual information* (MI) quantifies how much variability in the outcome functional limitations is captured by information on symptoms, signs and diseases. Total mutual information captures the combined predictive power of all three domains.

*Lost information* = captures the information lost if we exclude a specific exposure.

*Unique information* = refers to the amount of predictive power that a single domain (signs, symptoms or diseases) contributes on its own, independent of the other domains. If unique information is zero, it means every insight a given domain offers about functional limitations can be gleaned equally from other domains or only arises in combination with them.

*Pure Synergy* = The amount of information gained only when that domain is paired with at least one other domain.

*Synergy Lower Bound* = The guaranteed, minimal extra information from combinations involving that domain - essentially, what you definitely lose if you never allow it to interact with any other domain. Computed as the lost information minus the unique information.

*Synergy Upper Bound* = The maximal possible extra signal that could come from any combination of that domain with one, two, or all three of the other domains. It shows the ceiling on how much interaction-driven information that domain might contribute.

Percentages indicate the relative contribution of each domain in explaining functional limitations compared to the total explained variance (entropy = 100%). Higher percentages indic

ate a larger contribution.

Appendix 3. Sensitivity analyses repeating the Random Forest models using data from waves 1995/96 and 2021/22

|  | Diseases only  Mean Cohen’s κ (CI) | Diseases + Signs  Mean Cohen’s κ (CI) | Diseases + Signs + Symptoms  Mean Cohen’s κ (CI) |
| --- | --- | --- | --- |
| Wave 1995/96 |  |  |  |
| Total sample | 0·302 (0·225–0·405) | 0·304 (0·237–0·408) | 0·443 (0·341–0·524) |
| Multimorbidity subgroup | 0·166 (0·053–0·320) | 0·169 (0·023–0·343) | 0·370 (0·217–0·479) |
| Wave 2021/22 |  |  |  |
| Total sample | 0·282 (0·217–0·340) | 0·325 (0·242–0·408) | 0·525 (0·477–0·575) |
| Multimorbidity subgroup | 0·241 (0·102–0·352) | 0·291 (0·164–0·400) | 0·510 (0·406–0·593) |
|  | Symptoms only  Mean Cohen’s κ (CI) | Symptoms + Signs  Mean Cohen’s κ (CI) | Symptoms + Signs + Diseases  Mean Cohen’s κ (CI) |
| Wave 1995/96 |  |  |  |
| Total sample | 0·415 (0·321–0·506) | 0·414 (0·322–0·501) | 0·443 (0·341–0·524) |
| Multimorbidity subgroup | 0·369 (0·215–0·464) | 0·381 (0·246–0·492) | 0·370 (0·217–0·479) |
| Wave 2021/22 |  |  |  |
| Total sample | 0·494 (0·439–0·560) | 0·503 (0·448–0·560) | 0·525 (0·477–0·575) |
| Multimorbidity subgroup | 0·464 (0·402–0·525) | 0·447 (0·371–0·521) | 0·510 (0·406–0·593) |
|  | Signs only  Mean Cohen’s κ (CI) | Signs + Symptoms  Mean Cohen’s κ (CI) | Signs + Symptoms + Diseases  Mean Cohen’s κ (CI) |
| Wave 1995/96 |  |  |  |
| Total sample | 0·092 (0·053 – 0·142) | 0·414 (0·322 – 0·501) | 0·443 (0·341 – 0·524) |
| Multimorbidity subgroup | 0·131 (0·050 – 0·216) | 0·381 (0·246 – 0·492) | 0·370 (0·217 – 0·479) |
| Wave 2021/22 |  |  |  |
| Total sample | 0·077 (0·044 – 0·114) | 0·503 (0·448 – 0·560) | 0·525 (0·477 – 0·575) |
| Multimorbidity subgroup | 0·059 (0·032 – 0·091) | 0·447 (0·371 – 0·521) | 0·510 (0·406 – 0·593) |

In the original analyses, to optimise the computational power, data from waves 1995/96 to 2021/22 were treated as one cross-sectional dataset, not accounting for the within-person dependence of observations given the repeated measurements. In this sensitivity analyses, we reran the models using data from Waves 1995/96 (earliest included data collection wave) and 2021/22 (latest included data collection wave) to examine if not accounting for this dependency in observations affected the findings. The similar point estimates suggest little bias. The wider confidence intervals reflect the reduced number of datapoints and thus smaller computational power.

**References**

1.	Andresen EM, Malmgren JA, Carter WB, Patrick DL. Screening for depression in well older adults: evaluation of a short form of the CES-D (Center for Epidemiologic Studies Depression Scale). Am J Prev Med 1994;**10**:77-84.

2.	Zigmond AS, Snaith RP. The hospital anxiety and depression scale. Acta Psychiatr Scand 1983;**67**:361-370.

3.	Hunt SM, McEwen J, McKenna SP. Measuring health status: a new tool for clinicians and epidemiologists. J R Coll Gen Pract 1985;**35**:185-188.

4.	McEvoy JW, McCarthy CP, Bruno RM*, et al.* 2024 ESC Guidelines for the management of elevated blood pressure and hypertension. Eur Heart J 2024;**45**:3912-4018. <https://doi.org/10.1093/eurheartj/ehae178>

5.	Cruz-Jentoft AJ, Bahat G, Bauer J*, et al.* Sarcopenia: revised European consensus on definition and diagnosis. Age Ageing 2019;**48**:601. <https://doi.org/10.1093/ageing/afz046>

6.	Obesity: preventing and managing the global epidemic. Report of a WHO consultation. World Health Organ Tech Rep Ser 2000;**894**:i-xii, 1-253.

7.	World Health O. Waist circumference and waist-hip ratio : report of a WHO expert consultation, Geneva, 8-11 December 2008. Geneva: World Health Organization; 2011.

8.	Folstein MF, Folstein SE, McHugh PR. "Mini-mental state". A practical method for grading the cognitive state of patients for the clinician. J Psychiatr Res 1975;**12**:189-198.
